# Contribution of trehalose to ethanol stress tolerance of *Wickerhamomyces anomalus*

**DOI:** 10.1186/s12866-023-02982-y

**Published:** 2023-08-29

**Authors:** Yinfeng Li, Guilan Jiang, Hua Long, Yifa Liao, Liuliu Wu, Wenyue Huang, Xiaozhu Liu

**Affiliations:** 1 Guizhou Institute of Technology, Guiyang, 550000 People’s Republic of China; 2 Henan Institute of Science and Technology, Xinxiang, 453000 People’s Republic of China

**Keywords:** *Wickerhamomyces anomalus*, Ethanol stress, Trehalose, Transcriptomics, Antioxidant

## Abstract

**Background:**

The ascomycetous heterothallic yeast *Wickerhamomyces anomalus* (WA) has received considerable attention and has been widely reported in the winemaking industry for its distinctive physiological traits and metabolic attributes. An increased concentration of ethanol during ethanol fermentation, however, causes ethanol stress (ES) on the yeast cells. Trehalose has been implicated in improving survival under various stress conditions in microorganisms. Herein, we determined the effects of trehalose supplementation on the survival, differentially expressed genes (DEGs), cellular morphology, and oxidative stress tolerance of WA in response to ES.

**Results:**

The results indicated that trehalose improved the survival and anomalous surface and ultrastructural morphology of WA. Additionally, trehalose improved redox homeostasis by reducing the levels of reactive oxygen species (ROS) and inducing the activities of antioxidant enzymes. In addition, DEGs affected by the application of trehalose were enriched in these categories including in gene expression, protein synthesis, energy metabolism, and cell cycle pathways. Additionally, trehalose increased the content of intracellular malondialdehyde (MDA) and adenosine triphosphate.

**Conclusions:**

These results reveal the protective role of trehalose in ES mitigation and strengthen the possible uses of WA in the wine fermentation sector.

**Supplementary Information:**

The online version contains supplementary material available at 10.1186/s12866-023-02982-y.

## Introduction

The ascomycetous heterothallic yeast *Wickerhamomyces anomalus* (WA), or known as *Candida pelliculosa*, *Pichia anomala*, and *Hansenula anomala*, has gained considerable attention in recent years for its tolerance to diverse extreme environmental conditions [1]. This yeast has been reported in many different natural habitats, and it exhibits remarkable physiological robustness toward environmental stresses [2]. It can grow at a very low or very high pH (2.0–12.4) and at a temperature range of 3–37 °C, and it also has a high tolerance to osmotic stress (salt) and heavy metals [3, 4]. Moreover, WA shows wide-ranging antimicrobial activity against a variety of microorganisms as a result of the biosynthesis of volatile compounds or killer proteins, and it could be used for biopreservation [5].

Reportedly, WA has a broad carbon source capability and can produce ethanol from sucrose or xylose except for glucose [1]. Furthermore, WA secretes several kinds of glycosidases that can hydrolyze substrates containing glucosidic bonds and releases free glycoside ligands with aromatic characteristics. It has been widely used for food aroma improvement, especially in various winemaking processes [6]. Application of WA along with *Saccharomyces cerevisiae* as a mixed starter can positively influence the chemical composition and sensory features of produced cider [7]. Additionally, this mixed fermentation enriches the types and concentrations of the volatiles of kiwi wines and has better sensory properties when using WA along with *S. cerevisiae* [8]. The increased content of ethanol during ethanol fermentation introduces ethanol stress (ES) to the yeast cells, inhibits growth, increases death, and decreases the survival of the yeast cells [9, 10]. To date, the exact response mechanisms of WA to ES has remained obscure.

Trehalose, a nonreducing disaccharide, is commonly found in plants, microorganisms (yeast, bacteria, and fungi), and invertebrate animals [11–13]. Trehalose plays crucial physiological roles in organisms not only as a reserve carbohydrate but also as a protectant against different stresses [14]. Rehman et al. found that externally supplemented trehalose improves cadmium stress tolerance and mung bean (*Vigna radiata* L.) yield in both soil and hydroponic growth methods by attenuating cadmium uptake and improving photosynthetic efficiency and antioxidant activity [15]. In addition, trehalose confers protection to maize plants against salt stress and phosphorus deficiency [16]. Moreover, evidence has demonstrated that trehalose is involved in regulating the response of *S. cerevisiae* against osmotic stress, ES, dehydration stress, oxidative stress, thermal stress, and glucose starvation by preventing proteins from denaturing and that it suppresses the aggregation of denatured proteins [17–20].

In our previous research, we found that a high concentration of ethanol strongly suppressed the growth of WA and influenced nucleic acid metabolism, fatty acid metabolism, amino acid metabolism, and energy metabolism [21]. To reduce the adverse effects of ES on WA, we selected trehalose for application in this study. We investigated the survival, surface and ultrastructural morphology, antioxidant enzyme activities, reactive oxygen species (ROS) production, and gene expressions of WA to explore the roles of trehalose in the ES tolerance of WA. These findings provide a theoretical basis for further understanding the molecular basis of trehalose-induced ES tolerance in WA.

## Results

### Trehalose improves the survival of WA under ES

**Fig. 1:**
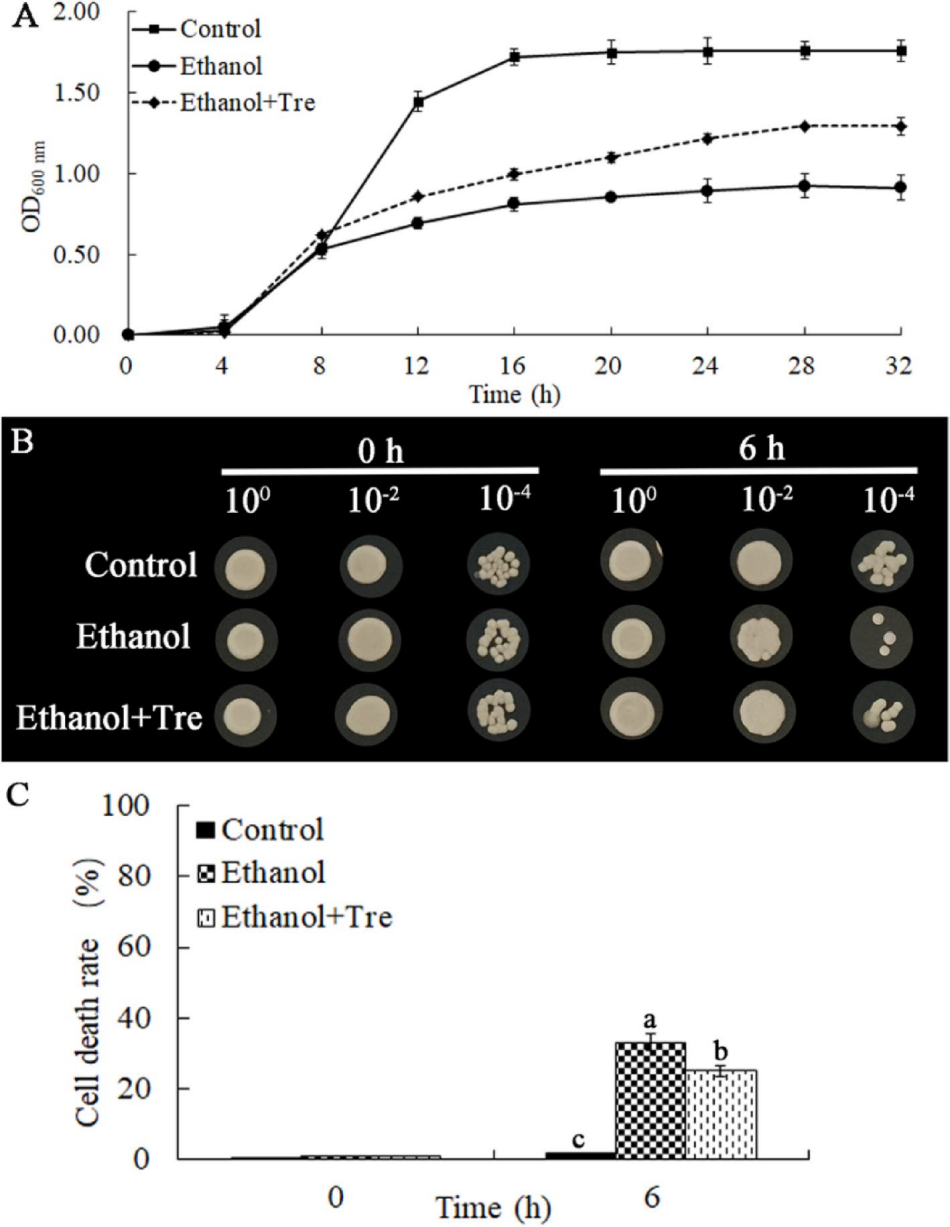
Trehalose could improve the survival of WA under ES. **A** Growth curves of WA; **B** Survival rate of WA; **C** Death rate of WA; Values in the same column labeled with distinct lowercase letters indicate significant differences (*P* < 0.05)

As displayed in Fig. 1A, the growth of WA was markedly suppressed with lower OD values compared to the control group when they were exposed to 9% ethanol. In contrast, trehalose could alleviate the suppressive effects of ES on the growth of WA by increasing the OD values.

When assessing the survival of WA cells, we found that the survival was inhibited, and the death increased after treatment with ethanol for 6 h (Fig. 1B). The decreased survival of WA cells was partially mitigated by the addition of trehalose. Furthermore, the addition of exogenous trehalose partially mitigated the heightened cell death rate induced by ES (Fig. 1C). In addition, the application of exogenous trehalose enhanced the compromised biomass caused by ES (Fig. S1).

### Ethanol induces trehalose accumulation in WA

**Fig. 2:**
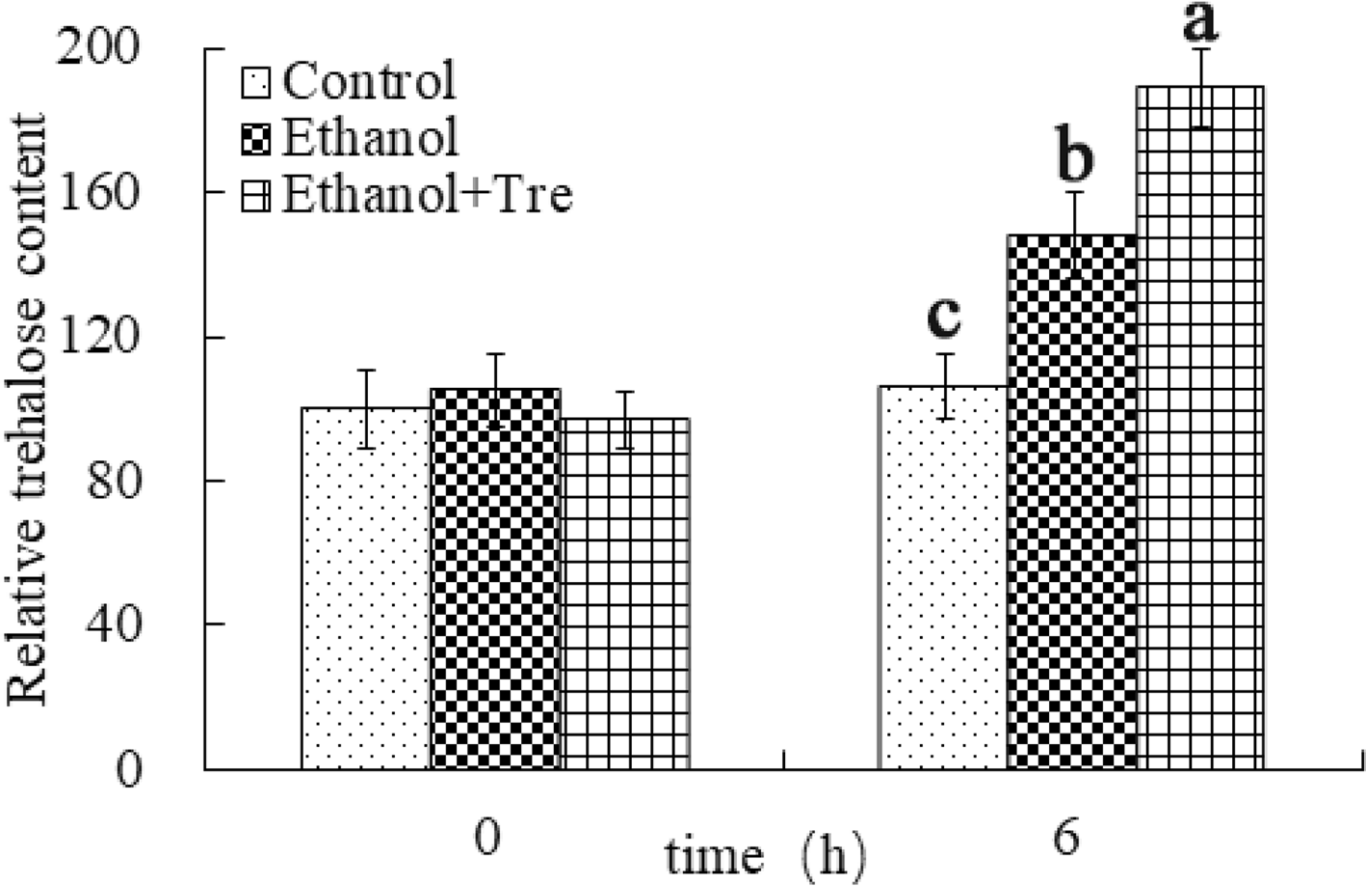
Relative content of trehalose in WA under ES. Values in the same column labeled with distinct lowercase letters indicate significant differences (*P* < 0.05)

As demonstrated in Fig. 2, the level of trehalose in each group was the same at the begining of the treatment. In contrast, the level of trehalose was remarkably elevated compared with the control group when exposed to ethanol for 6 h. These results indicated that ethanol induced trehalose accumulation, and trehalose might have played a role in the response to ES in WA. Moreover, exogenous application of trehalose led to a higher intracellular level of trehalose in WA during the entire ES period than that in cells without trehalose addition.

### Trehalose ameliorates the anomalous morphology of WA under ES

**Fig. 3:**
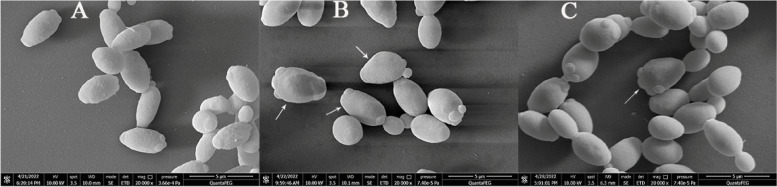
External morphological characteristics of WA. **A** Control group; **B** Ethanol group; **C** Ethanol + Tre group. Red arrows indicate morphology anomalous cells. Bar = 5 μm

We first examined the surface morphology of WA using a SEM. The external morphology of WA in the control group was ellipsoidal, full, and smooth (Fig. 3A). The surface of some WA cells became uneven and deformed, and the fullness decreased when exposed to ethanol (Fig. 3B). The anomalous external morphology of many WA cells, however, was restored to normal morphology after trehalose addition (Fig. 3C).

**Fig. 4:**
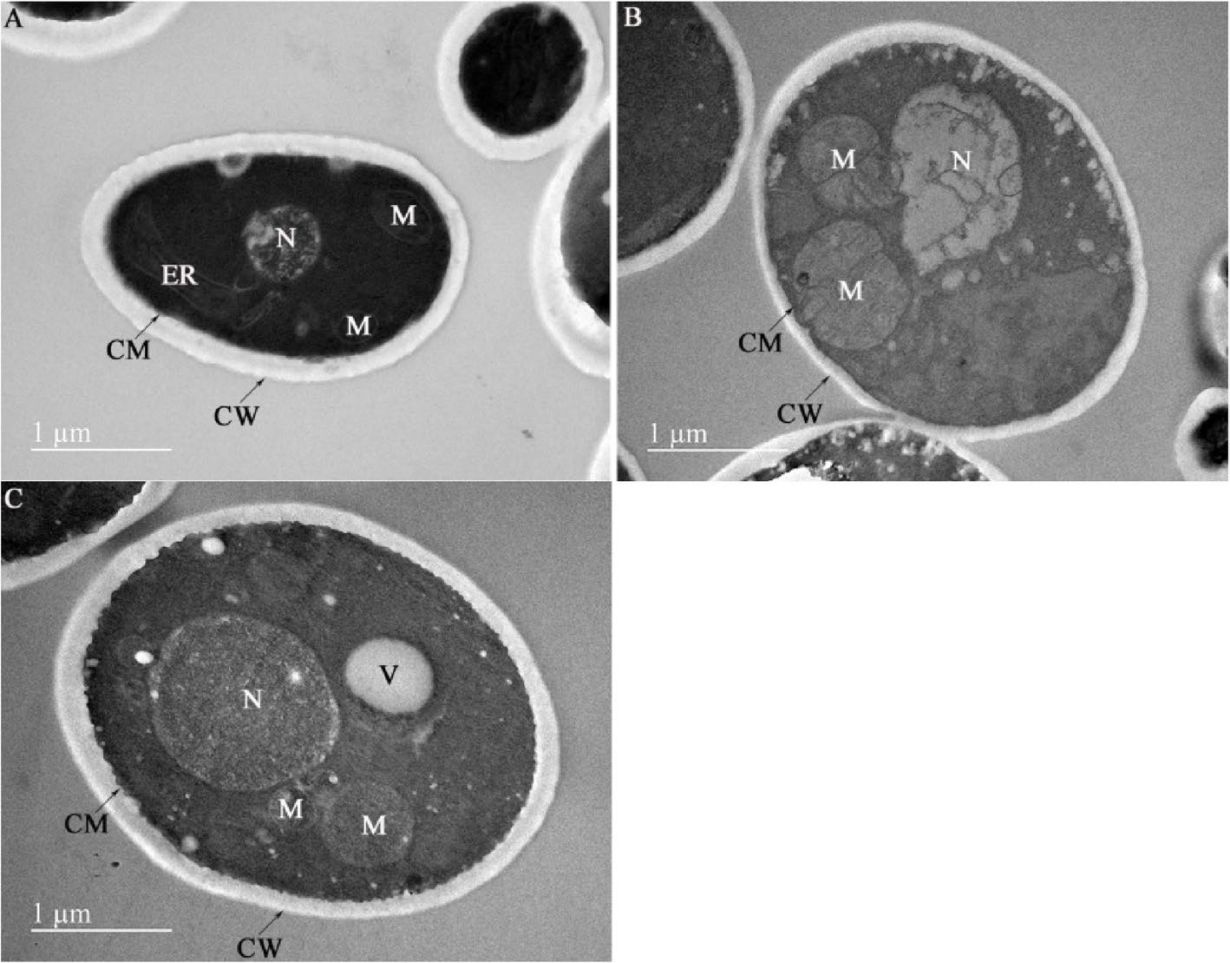
Cellular ultrastructure of WA under ES. **A** Control group; **B** Ethanol group; **C** Ethanol + Tre group. Bar = 1 μm. Arrows indicate the structure of cell membrane and wall. CM: cell membrane; CW: cell wall; V: vacuole; ER: endoplasmic reticulum; M: mitochondria; N: nucleus

We further verified the cellular ultrastructure of WA after replenishment of trehalose. Figure 4A shows that the nucleus, mitochondria, and endoplasmic reticulum of WA in the control group could be clearly observed, and the structures of the cell membrane, nucleus, and mitochondria were normal and had integrity. For the cells of the ethanol group, swelling of cytoplasmic organelles, especially for mitochondria, and a decline of cristae were the major characteristics of the ultrastructural morphology. The cell membrane was also destroyed by ethanol and became incomplete (Fig. 4B). Exogenous trehalose application alleviated the swelling of mitochondria and nucleus, which indicated that their function may have improved (Fig. 4C).

### Trehalose attenuates the oxidative stress and enhanced activities of antioxidant enzymes under ES

**Fig. 5:**
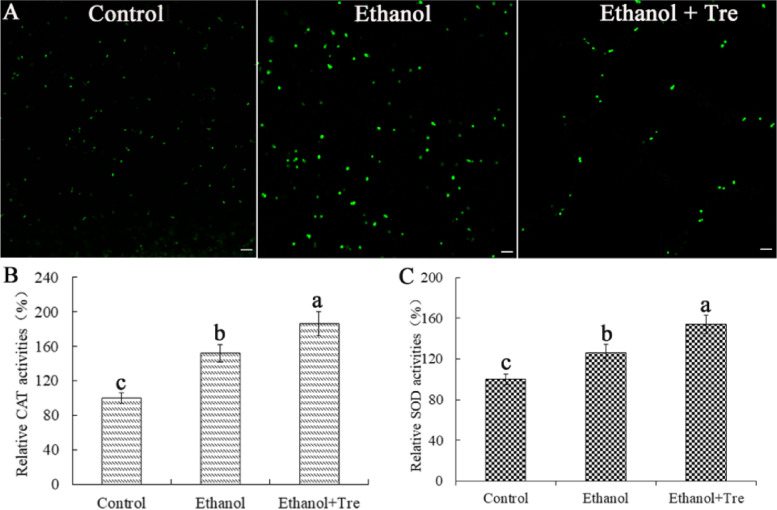
Trehalose intervention reduced ROS accumulation and enhanced antioxidant enzyme activities in WA under ES. **A** ROS levels of each group; **B** Relative CAT activities; **C** Relative SOD activities. Bar = 10 μm

As shown in Fig. 5A, the production of ROS was higher in ethanol group than in control group, indicating that the burst of ROS and the oxidative stress were induced by ES. The production of ROS, however, was reduced after trehalose intervention. Furthermore, the activities of antioxidant enzymes (e.g., CAT and SOD) were markedly influenced by ES in WA. As demonstrated in Fig. 5B and C, the activities of CAT and SOD were remarkably higher in ethanol group than in control group. Trehalose intervention also enhanced CAT and SOD activities, which were higher than in the ethanol and control groups. Clearly, exogenous trehalose application diminished oxidative stress by strengthening the activities of the antioxidant enzymes.

### Trehalose regulates the relative contents of malondialdehyde and adenosine triphosphate in WA

**Fig. 6:**
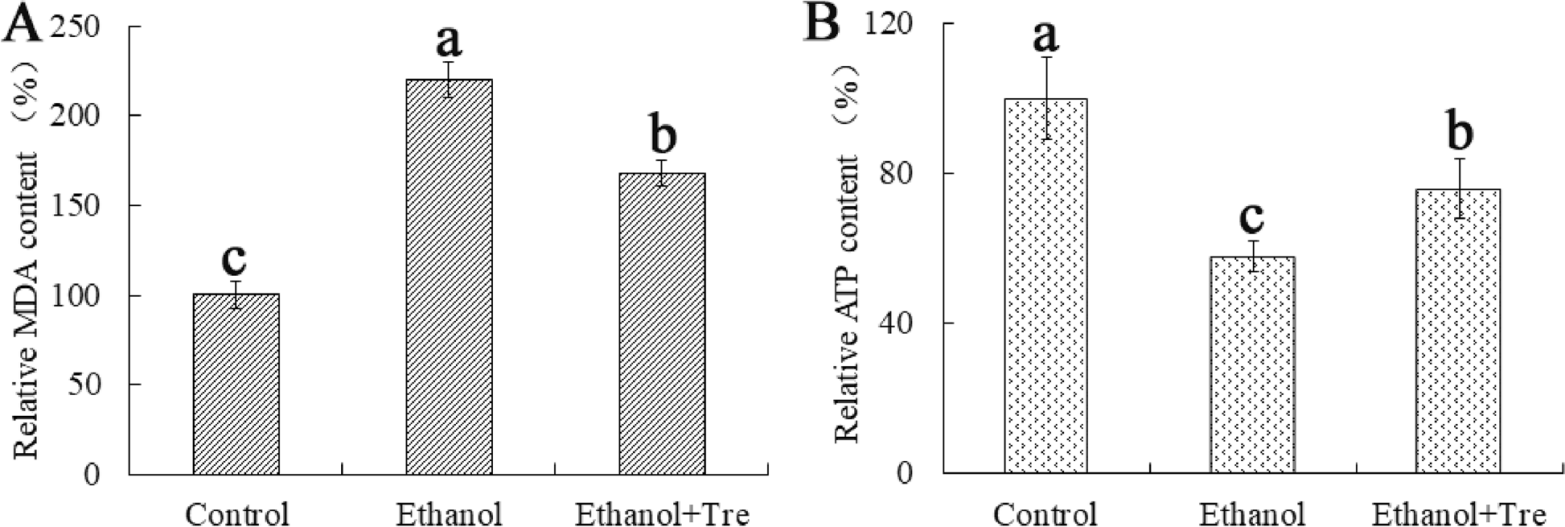
Intracellular levels of MDA (**A**) and ATP (**B**) in WA after trehalose intervention under ES

We determined the relative content of MDA, and the result showed that the MDA level was markedly increased in the ethanol group compared with the control group. In contrast, the relative content of MDA remarkably decreased after the addition of trehalose, suggesting that ethanol induced the oxidation of cell membranes and trehalose could reduce the degree of this oxidation (Fig. 6A). In addition, the results of ATP measurement (Fig. 6B) showed that the level of ATP was remarkably lower in ethanol group than in control group, suggesting that ES could decrease energy production because of the damaged mitochondria. The ATP level of ethanol + Tre group was dramatically higher than that of ethanol group, but was lower than that of control group. These findings indicated that ES could decrease the intracellular level of ATP, while trehalose exhibited an opposite trend.

### Trehalose regulated the transcriptome of WA under ES

#### Identification of differentially expressed genes (DEGs)

In total, we obtained 44,347,105.33, 43,247,100.67, and 43,713,072.67 raw reads in the control, ethanol, and ethanol + Tre groups, respectively. After data quality control, 43,022,481.33, 41,698,653.33, and 42,324,616.67 clean reads were acquired from these three sequencing groups, respectively. For all groups, the error rate was 0.03%, and both Q20 (%) and Q30 (%) values exceeded 95% (Table S1). The GC content was about 37%. Additionally, the results of principal component analysis (PCA) showed that the samples of the sequencing group were well separated from those of the other groups (Fig. S2). These data indicated that the sequencing samples' quality was deemed acceptable.

#### Definition of DEGs

**Fig. 7:**
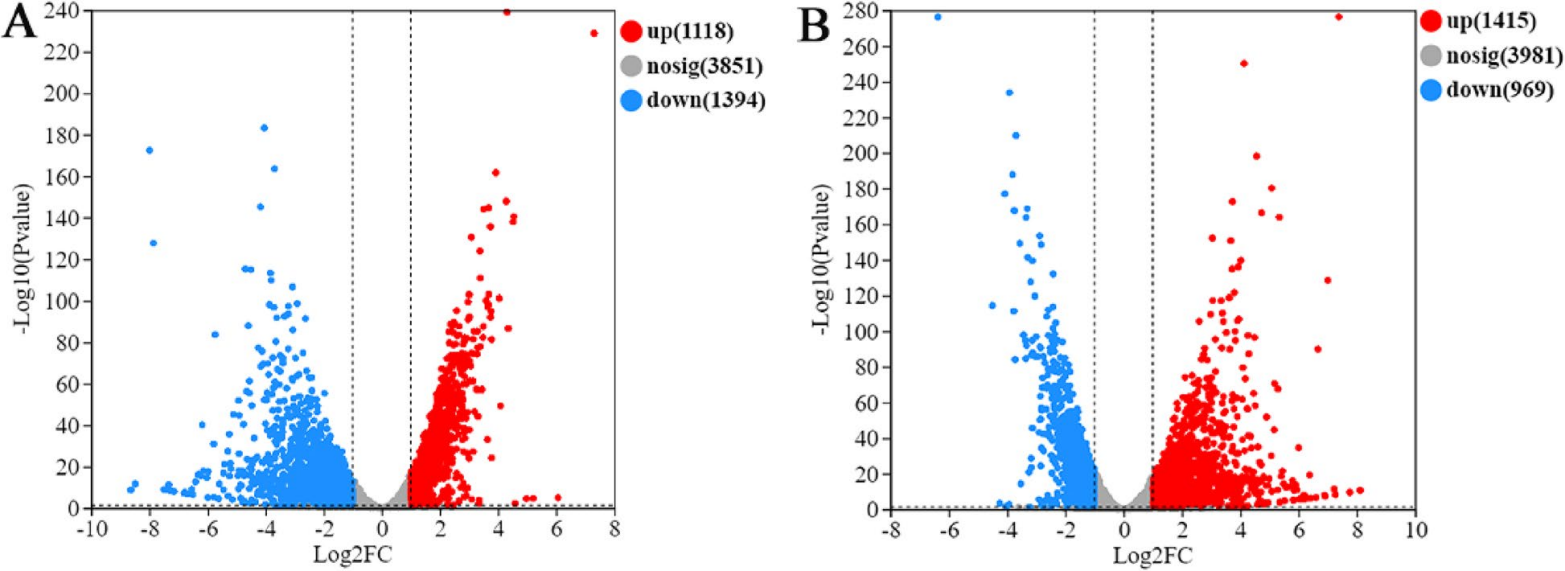
Volcano plot of DEGs after trehalose intervention under ES. **A** Volcano plot of DEGs between the ethanol and control groups; **B** Volcano plot of DEGs between the ethanol + Tre and ethanol groups

To further identify DEGs after trehalose intervention under ES, we used the following screening criteria: *P* < 0.05 and |log2(FC)|> 2. As shown in Fig. 7A, 2512 DEGs were significantly changed under stress for 6 h, including 1118 upregulated DEGs and 1394 downregulated DEGs. We found 2384 significantly changed DEGs, including 1415 upregulated DEGs and 969 downregulated DEGs, following exogenous supplementation with trehalose for 6 h under the ES condition (Fig. 7B).

#### Classification analysis of DEGs

**Fig. 8:**
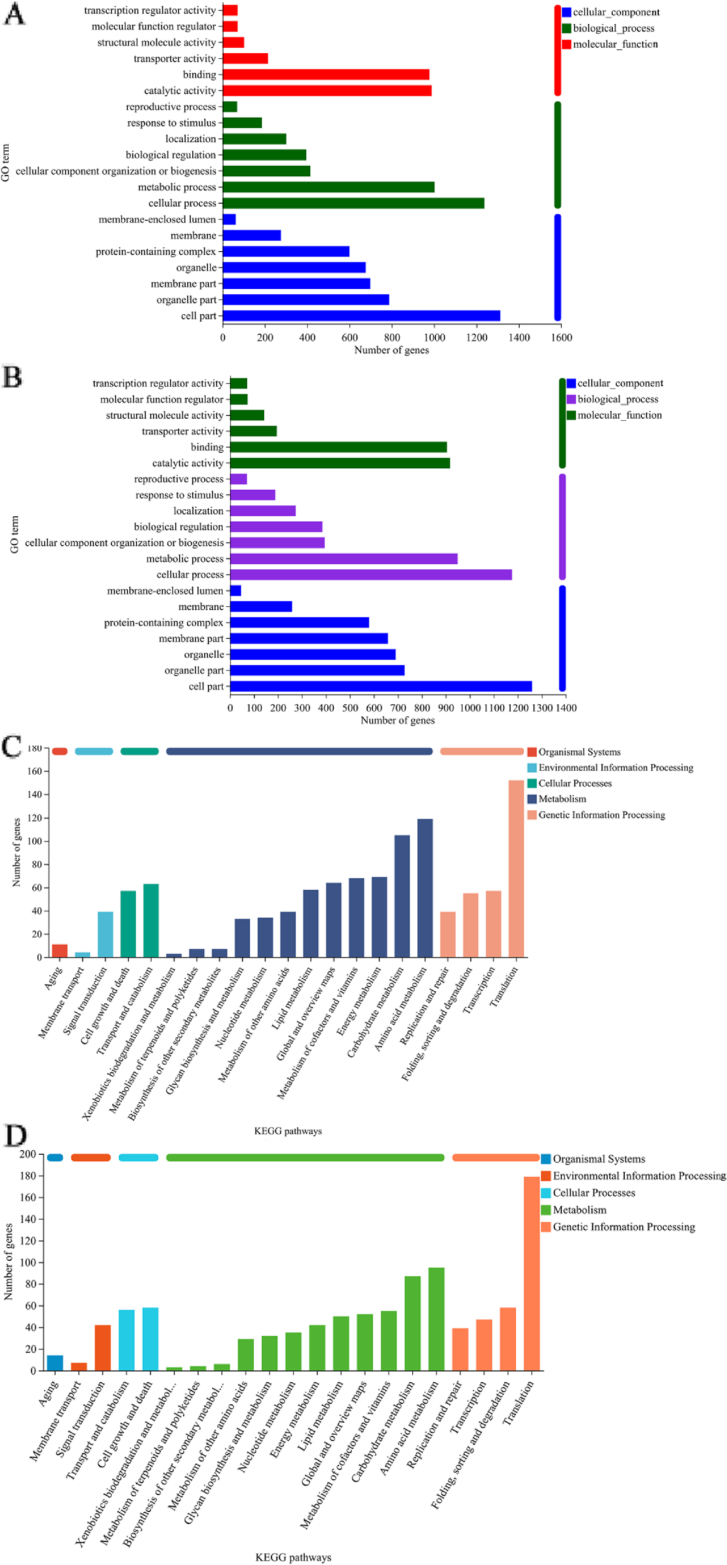
GO classification analysis and KEGG classification analysis of DEGs in WA after trehalose intervention under ES. **A** GO analysis of DEGs between the ethanol and control groups. **B** GO analysis of DEGs between the ethanol + Tre and ethanol groups. **C** KEGG analysis of DEGs between the ethanol and control groups. **D** KEGG analysis of DEGs between the ethanol + Tre and ethanol groups

To examine the potential function of these DEGs induced by trehalose intervention under ES, we performed Gene Ontology (GO) classification analysis and categorized the DEGs into 20 functional subcategories. The DEGs were classified according to 3 categories: molecular function, biological process, and cellular component. When WA cells were exposed to ethanol, most of the DEGs distributed in the categories of “molecular function”, “biological process”, and “cellular component” were annotated to “catalytic activity”, “cellular process” and “cell part”, respectively (Fig. 8A). These data suggested that the structure of the cells was destroyed and the intracellular metabolic reactions were disturbed when WA was stressed by ethanol. Note that the regulated targets of trehalose in WA cells have also been annotated to “cell part,” “cellular process,” and “catalytic activity” (Fig. 8B).

We further conducted KEGG analysis to check the potential functions of these DEGs induced by trehalose intervention under ES. We classified these DEGs into 5 categories: genetic information processing, metabolism, cellular processes, environmental information processing, and organismal systems. When analyzing the DEGs regulated by ES in WA, we found that “aging” was the sole term of the organismal systems, “signal transduction” accounted for most of the DEGs distributed in environmental information processing, and “transport and catabolism” accounted for most of the DEGs distributed in cellular processes (Fig. 8C). Amino acid metabolism was the most identified DEG in the term of “metabolism.” For the “genetic information processing” term, the most distributed DEGs were annotated to “translation.” When probing the DEGs induced by trehalose addition under ES in WA, the following terms also were annotated: “aging” in organismal systems, “transport and catabolism” in cellular processes, “signal transduction” in environmental information processing, and “translation” in genetic information processing (Fig. 8D).

#### KEGG analysis of differentially expressed genes

**Fig. 9:**
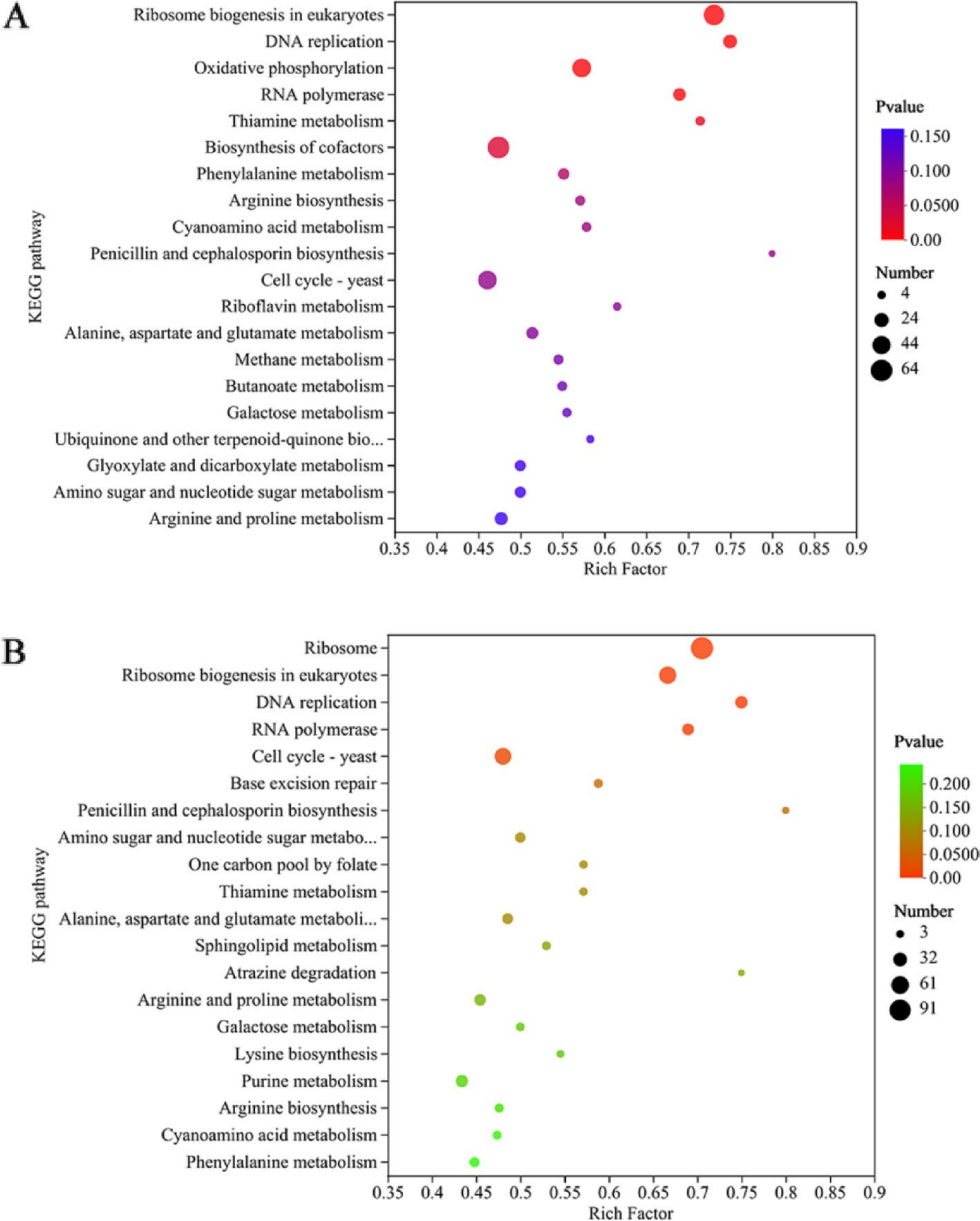
KEGG enrichment analysis of DEGs in WA after trehalose intervention under ES. **A** KEGG enrichment analysis of DEGs between the ethanol and control groups. **B** KEGG enrichment analysis of DEGs between the ethanol + Tre and ethanol groups

We further performed a KEGG analysis to explore the potential pathways in which the DEGs were involved. As shown in Fig. 9A, most of the DEGs induced by ethanol treatment were related to the following pathways: oxidative phosphorylation, meiosis-yeast, cell cycle-yeast, pyruvate metabolism, ribosome biogenesis, and biosynthesis of cofactors. These data indicated that ethanol influenced energy metabolism, protein synthesis, metabolic reactions, cell meiosis, and the cell cycle of WA cells.

After trehalose supplementation, these pathways, including ribosome, DNA replication, RNA polymerase, oxidative phosphorylation, glycolysis/gluconeogenesis, and cell cycle-yeast pathways, were abundantly enriched, suggesting that gene expression, protein synthesis, energy metabolism, and cell cycle were affected by trehalose addition (Fig. 9B).

## Discussion

In recent years, it has generally been accepted that non-*Saccharomyces* yeasts significantly affect the characteristics and aroma of wines. Among these yeasts, WA has attracted special attention in the wine industry because of its unique physiological and metabolic characteristics. It has been reported that WA is an interesting source of different enzymes, such as β-glucosidase, α-arabinofuranosidase, α-rhamnosidase, and β-xylosidase, which are involved in the release of aromatic compounds from the precursors and could be used in the winemaking industry [1]. Many authors have explored the application of this species along with *S. cerevisiae* in the fermentation of *Baijiu* [22], beer [23], rice wine [24], and fruit wines [25]. Fan et al. [26] proposed that the co-culture of WA and *S. cerevisiae* exhibited a favorable impact on ethyl-acetate formation, presenting potential opportunities to modify the aroma and flavor profile of Baijiu. In another study conducted by Zha et al. [27], the presence of WA in a mixed culture enhanced the flavor metabolism of *S. cerevisiae* during Baijiu production. The accumulated ethanol during ethanol fermentation, especially in the middle and late stages, exerted stress responses on the yeast cells, including reduced cell vitality, induction of yeast cell death, disruption of cell structure and function, modulation of gene expression, and subsequent decline in fermentation performance and efficiency [9, 10].

**Fig. 10:**
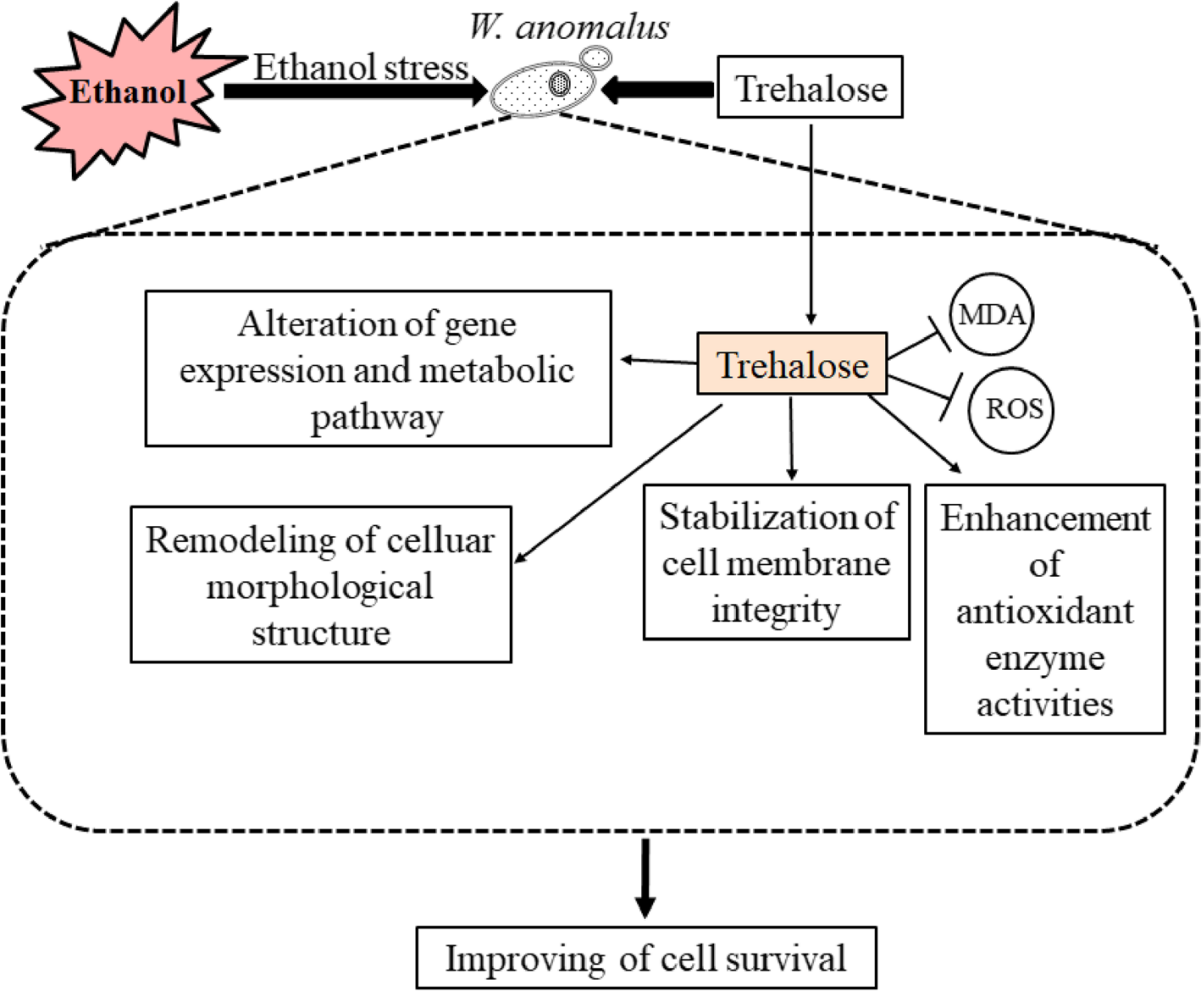
Proposed model depicting potential mechanisms through which trehalose facilitates the alleviation of ES in WA

Hence, the application of exogenous chemicals becomes essential to mitigate the damage inflicted by ES on yeast. Prior research has demonstrated that engineering the trehalose synthesis pathway offers a means to modulate yeast ES tolerance [28]. The ethanol yield of the engineered *S. cerevisiae* strain surpassed that of the wild-type strain, primarily attributable to the elevated intracellular trehalose accumulation resulting from *TPS1*(Trehalose-6-phosphate synthase) overexpression and *NTH1* (Neutral trehalase) deletion [29]. In addition, exogenous application of trehalose also promoted survival under ES condition [30]. In this study, the contribution of trehalose to ES tolerance of WA was investigated by exogenous trehalose supplementation, and our results clearly showed that trehalose served as a protectant against ES and that the exogenous supply of trehalose improved the survival of WA under ES (Figs. 1, 10).

Evidence shows that the increased concentrations of ethanol during fermentation prompt the generation of ROS [9, 10]. Excess ROS result in the occurrence of oxidative stress, which is cytotoxic to the cells, especially for cell organelles like DNA, proteins, and lipids [31]. In this research, we observed a higher level of ROS when WA was exposed to ethanol (Fig. 5A). In contrast, trehalose inhibited the generation of ROS. RNA-seq data showed that trehalose significantly regulated the DEGs annotated to DNA replication, ribosome, and RNA polymerase (Fig. 8B, D**)**. The antioxidant enzymes SOD and CAT have been shown to work synergistically to combat oxidative stress by scavenging excess ROS [32]. We also detected higher activities of SOD and CAT in WA after exogenous application of trehalose under ES (Fig. 5B, C**)**, which was consistent with a study on *S. cerevisiae* [33, 34].

The main targets of ethanol in yeast cells are thought to be plasma membranes [35]. An increased amount of ethanol during fermentation leads to lipid peroxidation, thereby affecting the permeability and fluidity properties of the plasma membranes [9, 10]. In this study, we found that the cell membrane was destroyed by ethanol when checking the ultrastructure of WA (Fig. 4A). Moreover, a higher level of MDA (lipid peroxidation product) was detected in ethanol group compared with control group (Fig. 6A), suggesting the lipid peroxidation by ethanol treatment in WA. In contrast, trehalose could mitigate this lipid peroxidation and stabilize the structural integrity of cell membranes. In addition, trehalose intervention also protected mitochondria against ES by ameliorating the swelling of mitochondria (Fig. 4C) and improving the structure of cristae, thus increasing the production of ATP (Fig. 6B).

The destroyed integrity of cell membranes may further disrupt the transport of amino acids into the cell [36]. Several amino acids have been found to have positive effects on ethanol tolerance in yeast, such as proline [37], arginine [38], and tryptophan [35]. In our previous study, we found that aspartate, glutamate, and arginine exhibited protective roles in WA cells against ES [21]. In this study, we also annotated alanine, aspartate, glutamate, proline, lysine, and phenylalanine metabolism from the transcriptomic data after the addition of trehalose (Fig. 9B). Thus, further studies on the mechanisms underlying the relationship between trehalose metabolism and the metabolism of these amino acids in the ethanol response of WA is warranted.

As a nonreducing disaccharide, trehalose acts as a stress protectant against various adverse environmental conditions, such as freezing, salinity, water, heat, and pinene, as well as oxidative and osmotic stress, but not limited to ES in microorganisms [11–13]. Most of the research on the protective roles of trehalose against these stresses has been performed using the baker’s yeast *S. cerevisiae* as a model. This approach, however, has several notable drawbacks. For example, some mutants were unable to synthesize trehalose for the disrupted metabolic pathway of trehalose, could not grow on readily fermentable sugars, and lost many regulatory responses. Several species of non-*Saccharomyces* yeasts have been used to explore the mechanism of yeast response to unlivable conditions. Péter and co-workers found that lipids and trehalose synergistically collaborated to manage heat stress in *Schizosaccharomyces pombe* [39]. This species of yeast also has been reported by Soto et al. [40], and they observed that the overexpression of *tps1* in *S. pombe*, leading to trehalose accumulation, resulted in enhanced resistance to multiple stresses, including osmotic stress and dehydration, freezing, and ES. In Dhanasekaran’s study, the results showed that trehalose supplementation improved the biocontrol efficiency of *Sporidiobolus pararoseus* via increased oxidative stress tolerance and altered transcriptome [41]. Additionally, Ma and co-workers [42] assessed the effects of trehalose and ergosterol on pinene stress of *Candida glycerinogenes*, and they found that overexpression of genes that encoded trehalose and ergosterol was related to tolerance to higher concentrations of pinene. In this study, we demonstrated the protective roles of trehalose in relieving ES to the yeast cells in WA because of the great potential for wine production. Moreover, the genomic sequence of WA had been finished and reported, which is convenient for research on the gene modification of this yeast species [2, 3]. In addition, WA could tolerate multiple adverse environmental conditions, such as extreme pH, high osmotic stress, and anaerobic conditions [2–5]. Therefore, this species of yeast also may be used as a model to probe the molecular mechanism of various stresses alongside ES [43, 44].

## Conclusions

To our knowledge, this study represents the first comprehensive analysis of the role of trehalose in contributing to ES tolerance in WA. Under ES, trehalose improved the survival and anomalous surface and ultrastructural morphology of WA. In addition, trehalose improved redox homeostasis by reducing the production of ROS and increasing the activities of antioxidant enzymes. In addition, the RNA-seq analysis provided evidence that trehalose altered the gene expression, protein synthesis, energy metabolism, and the cell cycle in WA, contributing to the proliferation and biological activities of yeast cells. These results revealed the protective role of trehalose in ES mitigation and strengthened the possible uses of WA in the wine fermentation sector.

## Materials and methods

### Yeast strains, media, and culture conditions

In this study, we usedstrain of WA C11, which was isolated from the spontaneous fermentation broth of *Rosa roxburghii* Tratt [45]. The yeast strain was cultured in YEPD medium for 72 h at 28 °C, and subsequently preserved at 4 °C for future use.

### ES conditions

WA C11 was cultured in YEPD broth medium for 8 h at 28 °C. We then assigned it into 3 groups: control group, ethanol group, and ethanol + Tre group. For the ethanol group, the cells were directly exposed to ES (9% v/v). The cells in the ethanol + Tre group were exposed to 9% ethanol and 10 mmol/L of trehalose. For the control group, the cells received neither ethanol nor trehalose treatment. The beginning of treatment was regarded as 0 h. After incubation for 6 h, we collected the cells from each group for the experiments of this study.

### Survival analysis of WA

We first determined the growth curve. The optical density (OD) values of the control, ethanol, and ethanol + Tre groups were determined at 600 nm every 4 h. We repeated each experiment in triplicate.

We conducted a spot analysis to further analyze the survival of yeast cells. After treatment, the cells were collected, washed, resuspended, and spotted onto YEPD solid plates according to the method of Liu and colleagues. Each colony was captured using a microscope (Olympus, Tokyo, Japan).

We then performed methylene blue staining to monitor the dead cells based on the method of Sami and co-workers [46]. The death rate was determined by counting the blue-stained cells, indicating dead cells, while the live cells remained unstained.

### Transcriptomic analysis of WA

We extracted total RNA from the yeast cells of the control, ethanol, and ethanol + Tre groups using TRIzol reagent (Invitrogen, USA) following the manufacturer’s protocols. We removed genomic DNA by digesting total RNA with DNase I (TaKara, Shiga, Japan). RNA-seq transcriptome library was established using a TruSeqTM RNA sample preparation kit (San Diego, CA, USA). cDNA target fragments of 300 bp were selected from the libraries using 2% Low Range Ultra Agarose, followed by PCR amplification with Phusion DNA polymerase (NEB, Ipswich, MA, USA) for 15 PCR cycles. The resulting paired-end RNA-seq sequencing library was then sequenced using the Illumina HiSeq xten/NovaSeq 6000 sequencer (Illumina, San Diego, CA, USA). We conducted the identification of DEGs, classification analysis of DEGs, and KEGG enrichment analysis of DEGs using the Majorbio Cloud Platform (https://cloud.majorbio.com) according to the method of Li and colleagues [47].

### Morphological analysis of WA

We assessed the surface morphological characteristics of WA using a scanning electron microscope (SEM) and observed the cellular ultrastructure using a transmission electron microscope (TEM). We collected the WA cells of the control, ethanol, and ethanol + Tre groups by centrifugation (4000 × g, 10 min), which were then rinsed 3 times with physiological saline. The cells were then suspended in 2.5% glutaraldehyde and underwent three washes with 0.1 M phosphate-buffered saline (PBS; pH 7.4). Subsequently, the cells were dehydrated with 0%, 50%, 70%, 85%, 95%, and 100% ethanol solutions. After drying with in a critical point dryer (Hitachi, Tokyo, Japan), WA cells were coated with a gold/palladium alloy, and then examined using an FEI Quanta FEG 450 SEM system (FEI, Hillsboro, OR, USA). The dehydrated WA cells were embedded in an epoxy resin and sectioned with a diamond knife (LKB-Nova, Elkin, NC, USA). The ultrathin sections were stained with lead citrate, viewed, and photographed with an FEI Tecnai spirit 120 kV (FEI).

### ROS determination

We measured the ROS levels of WA cells using the fluorescent dye dichlorodihydrofluorescein diacetate (DCFH-DA) [48]. After treatment, we collected the cells of each group by centrifugation (10,000 rpm, 10 min). The cells were rinsed with PBS (pH 7.4) three times and then stained with DCFH-DA (25 mM/L). We observed the ROS accumulation using a stereoscopic fluorescence microscope (Carl Zeiss Axio Zoom v16; Zeiss International, Oberkochen, Germany).

### Catalase and superoxide dismutase activity assay

We collected the cells by centrifugation (5000 rpm, 5 min) and rinsed them twice with PBS (pH 7.4). We determined the catalase (CAT) and superoxide dismutase (SOD) activities using the CAT and total SOD assay kits (Jiancheng Bioengineering, Jiangsu, China), respectively, following the manufacturer’s protocols. We calculated the activities of CAT and SOD according to the value of ethanol group or ethanol + Tre group divided by the that of control group.

### Measurement of trehalose, malondialdehyde, and adenosine triphosphate levels

We measured the levels of trehalose, malondialdehyde (MDA), and adenosine triphosphate (ATP) using the trehalose content detection (Jiancheng), MDA assay (Jiancheng), and ATP assay kits (Beyotime, Shanghai, China), respectively, following the manufacturer’s protocols. We calculated the levels of trehalose, MDA, and ATP based on the value of Eth group or ethanol + Tre group divided by that of control group.

### Statistics

All data are presented as mean ± standard deviation. We used SPSS 21.0 software (IBM-SPSS Inc., IL, USA) for the univariate analysis of variance (ANOVA) and determined the significance of the difference. *P* < 0.05 was deemed statistically significant.

### Supplementary Information

**Additional file 1:** **Figure S1.** Trehalose increased biomass of *W. anomalus* under ethanol stress. **Figure S2.** Principal component analysis (PCA) results of the samples used in this study. **Table S1.** Quality analysis of transcriptome sequencing data of different groups.

## Data Availability

All data generated or analyzed during this study are included in this published article and its supplementary information files.

## Abbreviations

*W. anomalus*: *Wickerhamomyces anomalus*
*S. cerevisiae*: *Saccharomyces cerevisiae*
DEGs: Differentially expressed genes
MDA: Malondialdehyde
ROS: Reactive oxygen species
CAT: Catalase
SOD: Superoxide dismutase
ATP: Adenosine triphosphate
RNA-seq: RNA-sequencing
PBS: Phosphatic buffer solution
OD: Optical density
PCR: Polymerase chain reaction
