# Supplementary material for: Contribution of trehalose to ethanol stress tolerance of Wickerhamomyces anomalus
Source: BMC Microbiol. 2023 Aug 29;23:239. doi: 10.1186/s12866-023-02982-y (PMC10463620; doi:10.1186/s12866-023-02982-y)
Supplement: Supplementary file 1 — Additional file 1: Figure S1. Trehalose increased biomass of W. anomalus under ethanol stress. Figure S2. Principal component analysis (PCA) results of the samples used in this study. Table S1. Quality analysis of transcriptome sequencing data of different groups. [file 12866_2023_2982_MOESM1_ESM.docx]

[Supplementary materials](javascript:;)


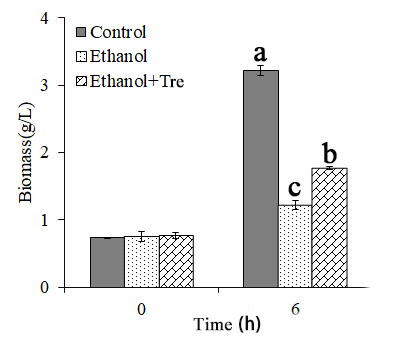


Figure S1 Trehalose increased biomass of *W. anomalus* under ethanol stress.


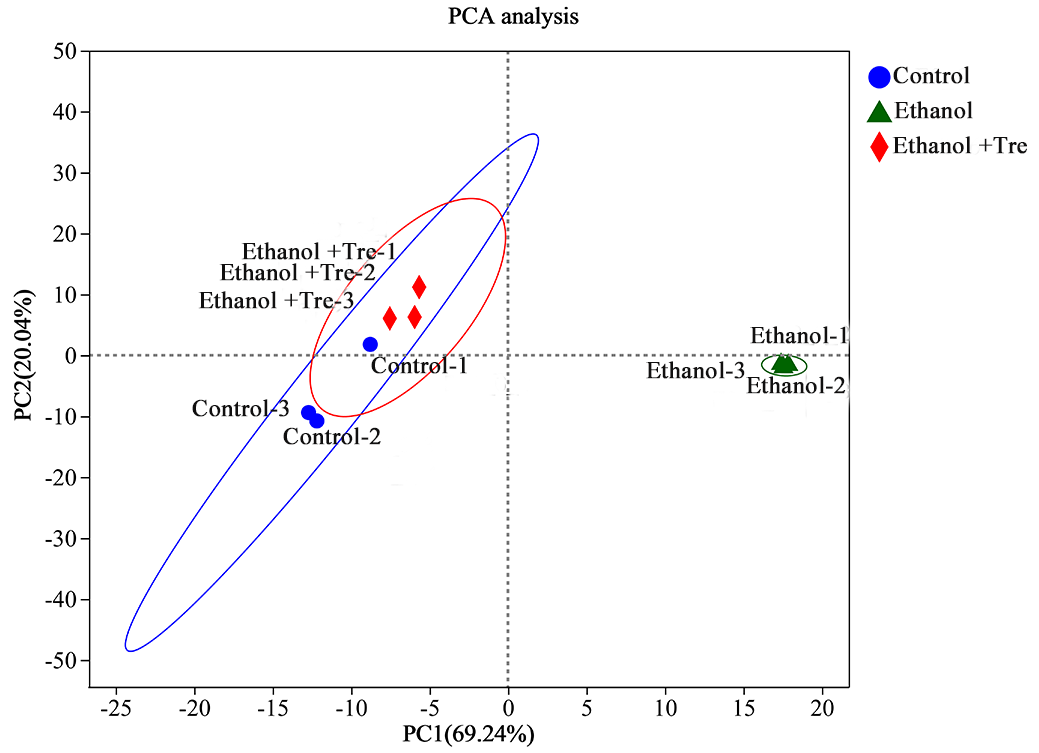


Figure S2 Principal component analysis (PCA) results of the samples used in this study

**Table S1** Quality analysis of transcriptome sequencing data of different groups

| **Group** | **Raw reads** | **Raw bases** | **Clean reads** | **Clean bases** | **Error rate (%)** | **Q20 (%)** | **Q30 (%)** | **GC (%)** |
| --- | --- | --- | --- | --- | --- | --- | --- | --- |
| Control | 44347105.33 | 6696412905 | 43022481.33 | 6386090702 | 0.03 | 97.98 | 93.85 | 37.69 |
| Ethanol | 43247100.67 | 6530312201 | 41698653.33 | 6197728299 | 0.03 | 97.75 | 93.37 | 37.49 |
| Ethanol+Tre | 43713072.67 | 6600673973 | 42324616.67 | 6274861869 | 0.03 | 97.84 | 93.56 | 37.85 |
